# Protective Role and Enhanced Intracellular Uptake of Curcumin in Retinal Cells Using Self-Emulsifying Drug Delivery Systems (SNEDDS)

**DOI:** 10.3390/ph18020265

**Published:** 2025-02-17

**Authors:** Elide Zingale, Sebastiano Masuzzo, Tatu Lajunen, Mika Reinisalo, Jarkko Rautio, Valeria Consoli, Agata Grazia D’Amico, Luca Vanella, Rosario Pignatello

**Affiliations:** 1 Department of Drug and Health Sciences, University of Catania, 95125 Catania, Italy; elidezingale@gmail.com (E.Z.); sebymasuzzo@gmail.com (S.M.); valeria_consoli@yahoo.it (V.C.); agata.damico@unict.it (A.G.D.); luca.vanella@unict.it (L.V.); 2 NANOMED—Research Centre for Nanomedicine and Pharmaceutical Nanotechnology, University of Catania, 95125 Catania, Italy; 3 CERNUT—Interdepartmental Research Centre on Nutraceuticals and Health Products, University of Catania, 95125 Catania, Italy; 4 School of Pharmacy, University of Eastern Finland, 70210 Kuopio, Finland; tatu.lajunen@helsinki.fi (T.L.); mika.reinisalo@uef.fi (M.R.); jarkko.rautio@uef.fi (J.R.); 5 Drug Research Program, Faculty of Pharmacy, University of Helsinki, 00100 Helsinki, Finland

**Keywords:** ocular drug delivery, nanoemulsion, eye, retina, diabetic retinopathy, Sirtuin-1, curcumin

## Abstract

**Background:** Sirtuin-1 (SIRT1), a histone deacetylase enzyme expressed in ocular tissues with intracellular localization, plays a critical protective role against various degenerative ocular diseases. The link between reduced SIRT1 levels and diabetic retinopathy (DR) has prompted the exploration of natural therapeutic compounds that act as SIRT1 agonists. Curcumin (CUR), which has been shown to upregulate SIRT1 expression, is one such promising compound. However, effective delivery of CUR to the deeper ocular tissues, particularly the retina, remains a challenge due to its poor solubility and limited ocular penetration following topical administration. Within this context, the development of self-nanoemulsifying drug delivery systems (SNEDDS) for CUR topical ocular delivery represents a novel approach. **Methods:** In accordance with our prior research, optimized SNEDDS loaded with CUR were developed and characterized post-reconstitution with simulated tear fluid (STF) at a 1:10 ratio, showing suitable physicochemical and technological parameters for ocular delivery. **Results:** An entrapment efficiency (EE%) of approximately 99% and an absence of drug precipitation were noticed upon resuspension with STF. CUR-SNEDDS resulted in a better stability and release profile than free CUR under simulated ocular conditions. In vitro analysis of mucoadhesive properties revealed that CUR-SNEDDS, modified with a cationic lipid, demonstrated enhanced interactions with mucin, indicating the potential for improved ocular retention. Cytotoxicity tests demonstrated that CUR-SNEDDS did not affect the viability of human corneal epithelial (HCE) cells up to concentrations of 3 μM and displayed superior antioxidant activity compared to free CUR in an oxidative stress model using retinal pigment epithelial (ARPE-19) cells exposed to hydroquinone (HQ). Cell uptake studies confirmed an enhanced accumulation of CUR within the retinal cells following exposure to CUR-SNEDDS compared to neat CUR. CUR-SNEDDS, at lower concentrations, were found to effectively induce SIRT1 expression. **Conclusions:** The cytocompatibility, antioxidant properties, and enhanced cellular uptake suggest that these developed systems hold promise as formulations for the delivery of CUR to the retina.

## 1. Introduction

Neurodegenerative eye diseases, including retinopathies, glaucoma, optic nerve atrophy, and age-related macular degeneration (AMD), primarily affect the posterior segment of the eye and are characterized by the activation of inflammatory and oxidative stress pathways [1]. Among them, retinopathy affects a significant portion of the population, with five million people worldwide suffering from DR. Under physiological conditions, the retina plays a crucial role in managing waste and providing protection against oxidative stress and inflammation [2]. Elevated glucose levels result in various malfunctions, including the downregulation of the SIRT1 protein, which diminishes its cytoprotective role within the tissue [3]. Sirtuins (SIRT1-SIRT7), a family of histone deacetylase proteins, play a crucial role in protecting tissues from degeneration, and SIRT1, particularly well-studied, regulates various cellular processes, including metabolism, apoptosis, cell differentiation, senescence, and genome stability, modulating gene expression [4,5]. In the context of retinal health, SIRT1 mitigates oxidative stress by reducing p53 acetylation, enhancing the activity of the antioxidant enzyme superoxide dismutase (SOD) and lowering the expression of vascular endothelial growth factor—α (VEGF-α) in diabetic retinas. Additionally, it regulates inflammatory processes by modulating key genes, including interleukins (IL-1, IL-8, and IL-6) and factors such as NF-κB (nuclear factor kappa, a light-chain enhancer of activated B cells) and TNFα (tumor necrosis factor) [6,7,8]. Various natural molecules, particularly polyphenolic compounds, enhance SIRT1 activity by reducing the substrate concentration required for a half saturation of the enzyme, thereby decreasing the amount of substrate needed for full enzymatic activity [9]. CUR, primarily found in *Curcuma longa*, is a naturally abundant compound classified as a SIRT1 agonist with potential therapeutic applications in the management of DR due to its potent anti-inflammatory and antioxidant effects. It counteracts high-glucose-induced downregulation of SIRT1 by modulating key transcription factors [10]. CUR activates AMP-activated protein kinase (AMPK), thereby increasing the NAD+/NADH ratio, which is essential for SIRT1 enzymatic function [10]. This activation enhances SIRT1 activity, promoting the deacetylation of the key transcription factors and enzymes involved in the oxidative stress response, inflammation, and metabolic regulation [11,12,13]. CUR exhibits anti-apoptotic effects by inducing heme oxygenase-1 (HO-1) through the Nrf2 pathway, which upregulates SIRT1 expression and strengthens antioxidant defenses [14]. Its ability to inhibit NF-κB-mediated inflammatory pathways, along with its ROS scavenging properties, enhances the body’s defenses in disease conditions such as DR and AMD [15]. In the DR scenario, CUR reduces VEGF expression, regulates angiogenesis, and influences CaMKII/NF-κB and peroxisome proliferator-activated receptor-γ (PPAR-γ) signaling pathways to mitigate leukocyte infiltration and oxidative stress [16,17]. It also improves glucose tolerance by enhancing the secretion of glucagon-like peptide 1 (GLP-1), addressing both hyperglycemia and hyperlipidemia [18]. To better understand SIRT1 pathways for protection against oxidative stress and inflammation, different studies showed exhaustive graphics that can be seen in [19,20].

Despite its therapeutic potential, CUR clinical application is hindered by poor bioavailability, rapid metabolism, and low aqueous solubility. Notably, cellular membrane penetration is limited due to its poor permeability [21]. With the aim of targeting the upregulation of SIRT1, the poor permeability of CUR is a critical factor to consider due to the intracellular localization of the protein in retinal cells. These challenges highlight the need for innovative delivery systems to enhance CUR penetration. Moreover, the presence of tautomerism, particularly keto–enol equilibrium, influences its chemical reactivity, stability, and solubility. CUR exhibits pH-dependent behavior and is inherently unstable, especially at physiological pH (7.4). Under acidic conditions, CUR remains stable, whereas at a neutral or alkaline pH, it undergoes hydrolysis, leading to the formation of ferulic acid, vanillin, and other degradation products. The design of SNEDDS represents a promising approach to address CUR-associated limitations. SNEDDS is a nanotechnological formulation essentially based on an isotropic mixture of oil, surfactant, and co-surfactant that spontaneously forms a heterogeneous nanoemulsion upon contact with an aqueous medium, such as tear fluid, under mild agitation and specific environmental conditions, including pH and temperature [22]. SNEDDS have been extensively studied for the oral delivery of lipophilic drugs, including CUR, in various pharmaceutical forms. They offer a high solubilization capacity for a wide range of drug compounds with varying degrees of lipophilicity [23,24]. Few studies have focused on the application of SNEDDS in the ocular field, making these systems still underexplored in this context [25,26,27]. Furthermore, despite the extensive literature on CUR, the formulation of SNEDDS for the topical ocular delivery of CUR in the treatment of DR is a novel approach that has not yet been explored. The drug can be maintained in a solubilized form, increasing the dissolution rate of poorly water-soluble drugs and increasing the apparent solubility in water. This formulation could also help to enhance absorption and improve passage through the ocular barriers.

This paper will explore CUR-loaded SNEDDS (CUR-SNEDDS) developed by utilizing preformulation studies from our previous work, in which the design of experiments (DoE) approach was employed during the optimization phase to design an appropriate formulation for topical ocular delivery [22]. The objective of the formulation is to improve CUR’s cellular permeability and therapeutic effects in the eye, aiming to maximize its protective benefits against the complex pathological processes of DR. CUR-SNEDDS were designed based on the selection of optimal materials with the highest CUR solubility for SNEDDS preparation. Building upon the optimized formulation from previous studies [22], a CUR-loaded SNEDDS-based formulation was prepared and characterized in terms of particle size, polydispersity index (PDI), zeta potential (ZP), pH, osmolarity, and percentage of transmittance. Additionally, various technological parameters, including morphology, viscosity, emulsion time, and EE%, were assessed. The drug release profile was evaluated through in vitro studies simulating a physiological environment. Furthermore, the cytocompatibility of CUR-SNEDDS was tested in ARPE-19 cells and HCE to assess cytocompatibility for both topical administration and retinal targeting. To evaluate the formulation’s potential to penetrate the cell membrane and reach the SIRT1 target, cellular uptake was examined using fluorescence microscopy in ARPE-19 cells. Furthermore, CUR-loaded SNEDDS’ capacity for inducing SIRT1 protein expression was assessed. Finally, the formulation’s ability to mitigate oxidative stress was assessed by evaluating cellular viability and recovery after oxidative stress induction using HQ.

## 2. Results

### 2.1. CUR Solubility in Different Vehicles

**Figure 1:**
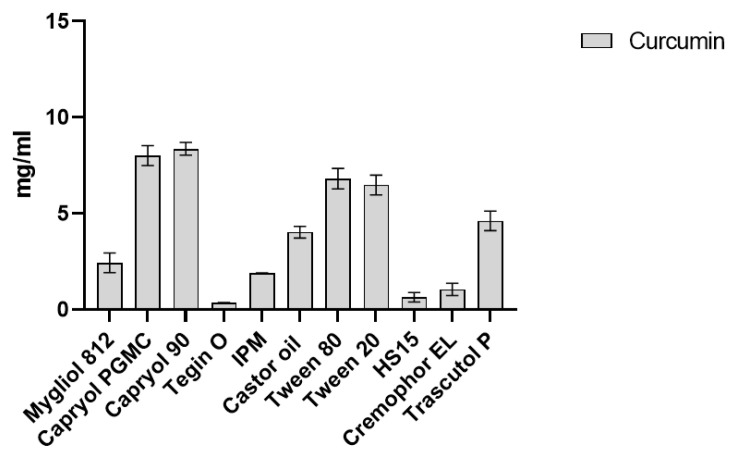
Solubility (mg/mL) of CUR in different vehicles (oils and surfactants).

CUR solubility was determined in various oils (Mygliol^®^ 812, Capryol^®^ PGMC and Capryol^®^ 90, Tegin^®^ O, and isopropylmyristate (IPM), Castor oil^®^) and surfactants (Tween^®^ 20, Tween^®^ 80, Cremophor^®^ EL, Solutol ^®^HS15, and Transcutol^®^ P), as shown in Figure 1. CUR was more effectively solubilized in Capryol^®^ 90 and Capryol^®^ PGMC, achieving concentrations of 8.36 mg/mL and 8 mg/mL, respectively. Among the surfactants tested, CUR was best solubilized in Tween^®^ 80 at a concentration of 6.80 mg/mL and in Transcutol^®^ P at 4.61 mg/mL. The vehicles are presented in descending order of solubilizing capacity: Capryol^®^ 90 > Capryol^®^ PGMC > Tween^®^ 80 > Tween^®^ 20 > Transcutol^®^ P > Castor oil > Miglyol^®^ 812 > IPM > Cremophor^®^ EL > Solutol^®^ HS 15 > Tegin^®^ O. Based on this solubility assessment, the materials to be used as starting points were selected. In a previous study by Zingale et al., four SNEDDS-based formulations were optimized using DoE software. Among the optimized formulations, one composed of Capryol^®^ PGMC, Tween^®^ 80, and Transcutol^®^ P demonstrated superior stability [22]. This formulation, previously referred to as “Formulation A”, was selected for CUR loading due to the optimal solubility of CUR in these excipients. Therefore, Formulation A, consisting of Capryol^®^ PGMC, Tween^®^ 80, and Transcutol^®^ P at the concentrations specified in Table 1 in Section 4.3, was identified as the most suitable SNEDDS formulation for the development of CUR-loaded SNEDDS.

### 2.2. Physicochemical Characterization

**Figure 2:**
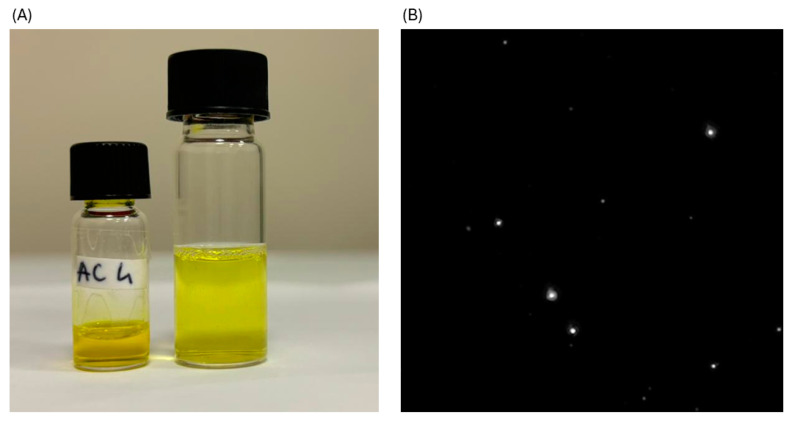
(**A**) Macroscopic visualization of AC and AC after reconstitution 1:10 in STF and (**B**) microscopic morphological analysis of AC after reconstitution (1:100,000 with PBS) by Zeta view analysis.

The unloaded formulation (A) and the CUR-loaded formulation (AC) were prepared and characterized based on physicochemical parameters to assess their suitability for topical ocular administration, specifically in terms of particle size, surface charge, homogeneity, clarity, and emulsification time. In addition, a cationic CUR-SNEDDS formulation (AC+) was prepared by incorporating didodecyldimethylammonium bromide (DDAB) and characterized using the same parameters. The incorporation of DDAB aimed to enhance retention time on the ocular surface through electrostatic interactions with the negatively charged mucin in the mucus layer. Upon resuspension at a 1:10 ratio in STF, all formulations exhibited a particle size of less than 50 nm. The resuspension in a small volume of STF with a ratio of 1:10 (SNEDDS: STF) more accurately simulated the resuspension on the ocular surface after topical administration, where only a very low volume is available for SNEDDS reconstitution [22]. The PDI was below 0.3, indicating a high degree of homogeneity. Visually, all three formulations demonstrated enhanced apparent solubility of CUR, appearing as clear solutions without precipitation and exhibiting transmittance values ranging from 99% to 100%. The formulation was designed to have a high solubilizing capacity, enabling the incorporation of the drug dose in a minimal volume while providing protection against degradation, thereby enhancing the compound’s bioavailability. Figure 2A shows anhydrous AC and AC after 1:10 reconstitution with STF. Notably, after reconstitution, the formulations AC and AC+ exhibited no precipitates, confirming the excellent incorporation of CUR within the formed globules. The application of SNEDDS technology significantly enhanced the solubility of CUR in water, increasing it from an inherent solubility of <1 μg/mL to an apparent solubility of 100 μg/mL—an approximately 100-fold improvement compared to raw CUR. The ZP was approximately neutral at −4 mV, as no component imparted a significant charge to the system. Non-ionic surfactants typically produce a slightly negative interface charge at neutral pH due to the differential adsorption of hydroxyl ions (OH⁻) and hydrated oxonium ions (H₃O⁺). The incorporation of DDAB at a final concentration of 0.25% in the reconstituted 1:10 formulation reversed the charge, yielding a slightly positive ZP of +2.92 ± 0.09 mV. The emulsification time was less than one minute and increased from formulation A to the loaded formulations. The preliminary morphology of the AC formulation was evaluated using microscopy combined with size measurement techniques (Figure 2B).

As shown in Figure 2B, very small spherical globules with a particle concentration of 5 × 10^7^ particles/mL were observed. The formulation also demonstrated ocular compatibility in terms of isotonicity, with a pH of 7.16 ± 0.12 for AC and 7.01 ± 0.05 for AC+ following reconstitution in STF. The viscosity of the formulation increased with shear stress, maintaining a range between 10 and 15 mPa·s. This viscosity remained largely constant despite the increasing shear stress, exhibiting the typical behavior of Newtonian fluids. This finding is particularly significant because shear forces, such as blinking or ocular drainage, could potentially impact the formulation. However, the formulation’s resistance to shear stress suggests it would remain stable under these conditions.

### 2.3. Mucoadhesive Properties of Cationic CUR-SNEDDS

**Figure 3:**
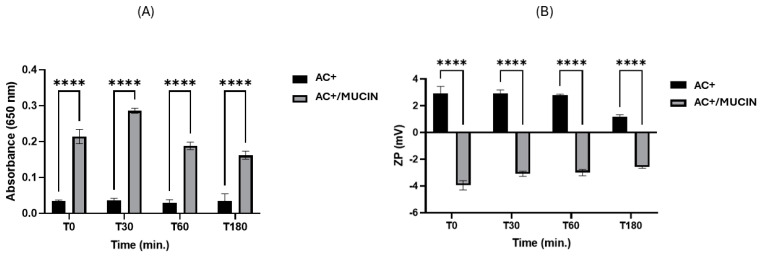
Mucoadhesion strength of A+ in contact with mucin dispersion in terms of (**A**) absorbance and (**B**) zeta potential. Each bar represents the mean value ±SD; *n* = 3. Statistical analysis was performed by 2-way ANOVA (**** *p* < 0.0001 vs. A+ at different time points).

Absorbance analysis via UV spectrophotometry and ZP measurements were performed on the cationic formulation A+ at various time points during contact with mucin. The absorbance of formulation A remained consistently low throughout the testing period, at approximately 0.1. Upon contact with a mucin dispersion, the absorbance of the A+/mucin sample initially increased, reaching a peak within 30 min before subsequently decreasing to a value lower than the initial measurement, though still significantly higher than that of A+ alone. The A+ samples exhibited a gradual increase in absorbance over time (Figure 3A) in the presence of mucin, likely due to electrostatic interactions that enhanced contact between mucin molecules and charged globules, thereby increasing sample turbidity. Notably, significant changes in ZP (Figure 3B) were observed, reflecting interactions between oppositely charged components, with a tendency for the ZP to decrease. It is noteworthy that mucin significantly influenced these interactions, almost acting as a coating for the SNEDDS globules. Considering the ZP of the mucin dispersion alone, which was approximately −6.82 mV throughout the analysis period, the ZP of formulation A+ became less negative upon contact with mucin and showed a trend of gradual increase over time.

### 2.4. Drug Entrapment and Release

**Figure 4:**
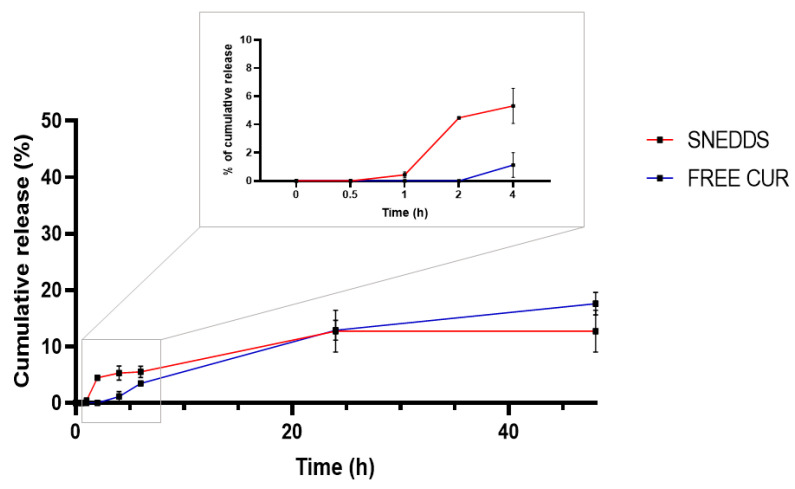
In vitro CUR release from CUR-SNEDDS (AC) compared to free CUR investigated for 48 h.

SNEDDS retains approximately 99% of CUR, as indicated by the absence of precipitation during resuspension and the clear formulation, which exhibited no opacity. The dialysis bag method was employed for in vitro release studies of SNEDDS to address this challenge. A mixture of methanol/Tween^®^ 80/water (30:10:60, *v*/*v*/*v*) was used as the external medium. A slow release of CUR was observed over 48 h, with a plateau being reached at 24 h, followed by controlled release until 48 h. The release of CUR from SNEDDS is attributed to the spontaneous formation of a nanoemulsion due to the low surface free energy at the oil–water interface, which enables immediate solubilization of the drug in the release medium with specific properties. This explains why more than 10% of CUR is released within the first 24 h. It is important to note that, despite the slow release, the release of CUR from SNEDDS is significantly greater than that of free CUR (Figure 4). During self-emulsification, when SNEDDS comes into contact with an aqueous fluid, the components of the nanoemulsion (oil, surfactant, and co-surfactant) effectively form globules with a narrow size distribution. This process increases the surface area and reduces surface free energy, ultimately enhancing the drug release rate. These factors collectively improve the dissolution, permeability, and release of CUR [28].

### 2.5. Stability

**Figure 5:**
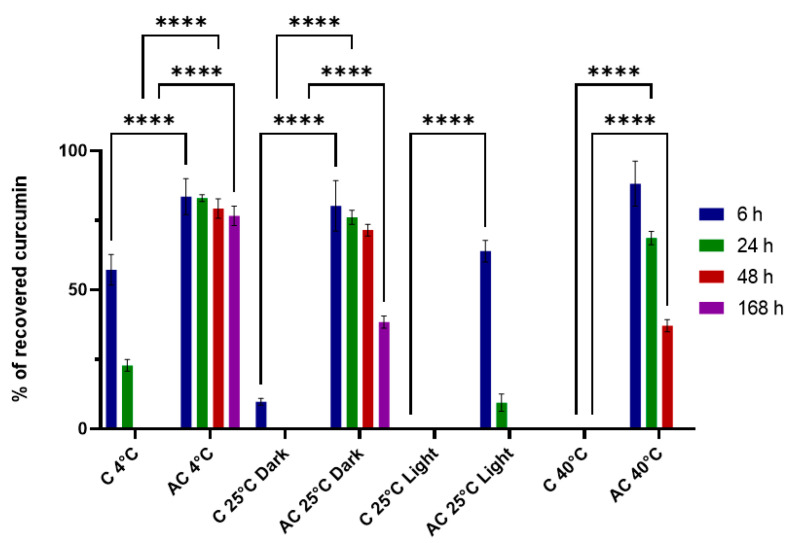
Stability investigation of native CUR(C) in PBS and CURC-loaded SNEDDS (AC) at different conditions of exposition: 4 °C, 25 °C light and dark, and 40 °C. (Statistical analysis was made with Tukey’s multiple comparisons test **** *p* < 0.0001 vs. C at different exposition conditions).

Native CUR was observed to undergo rapid degradation in phosphate buffer saline (PBS), with only 9% remaining after 6 h of incubation at room temperature (r.t.) in the dark. In contrast, CUR encapsulated within CUR-SNEDDS remained stable under the same conditions, with 80% of CUR recovered (Figure 5). Significant differences were evident as early as 6 h under various exposure conditions. For example, CUR in PBS at 4 °C retained 57% of its original amount, whereas the AC formulation retained 87%. At 25 °C in the dark, CUR in PBS degraded to less than 10% within 6 h, while CUR encapsulated in SNEDDS was preserved with a recovery of 80.2%. Further, CUR in PBS exposed to light at 25 °C and to 40 °C in the dark was completely degraded after 6 h, rendering it unquantifiable. In contrast, the AC formulation demonstrated recoveries of 64% and 88% under the same conditions, respectively, indicating excellent protection of CUR within the SNEDDS formulation. Long-term stability studies showed that the formulation retained better stability at 4 °C, with recoveries of 83%, 79%, and 77% at 24, 48, and 168 h, respectively. A time-dependent degradation trend was observed at 25 °C in the dark, with CUR recovery decreasing to 38% after 168 h, whereas free CUR in PBS was completely degraded after 6 h. Under light exposure at 25 °C, the CUR within the SNEDDS formulation was protected for up to 24 h. This protective effect was particularly pronounced at 40 °C, where CUR remained quantifiable, with a recovery of 37% after 48 h of exposure. These findings suggest that CUR is more susceptible to light-induced degradation than heat, and they underscore the significant protective effect of the SNEDDS formulation compared to free CUR. In summary, the formulation notably enhanced the stability of CUR in PBS, effectively protecting the encapsulated CUR against degradation. A table in the Supplementary Materials resumes the above findings (Table S1).

### 2.6. Cytocompatibility Studies

**Figure 6:**
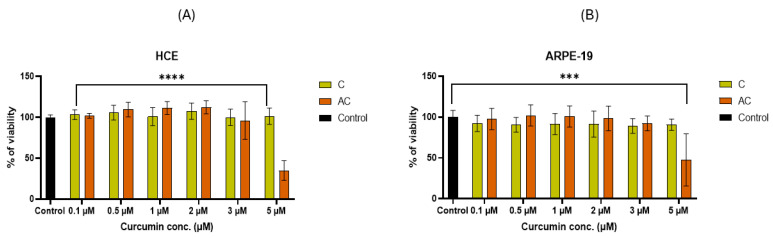
Evaluation of cytotoxicity of CUR-SNEDDS loaded with different concentrations of CUR (0.1–5 μM), respectively, on the (**A**) HCE and (**B**) ARPE-19 cell lines (**** *p* < 0.0001 vs. control, *** *p* < 0.0005 vs. control).

Cytocompatibility studies on the formulation were conducted using two different cell lines: a human corneal cell line, HCE, and a human retinal pigment epithelial cell line, ARPE-19. The purpose of this dual evaluation was to assess the cytocompatibility of the formulation with the ocular and corneal surface, the site of administration, and the retina, the target site. The AC formulation demonstrated cytocompatibility at all tested concentrations, except for the 5 μM concentration, which caused a significant reduction in cell viability compared to the control. Specifically, a 34% reduction in viability was observed in HCE cells (Figure 6A), while ARPE-19 cells exhibited a 47.6% reduction (Figure 6B). This reduction in viability could be attributed to an increased cellular uptake of CUR, which may result in toxicity for both cell lines, thereby reducing cell viability. Notably, this effect was not observed with free CUR, as it has a limited ability to cross the cell membrane in the absence of a carrier.

### 2.7. Curcumin Uptake in ARPE-19 Cells and SIRT1 Expression

**Figure 7:**
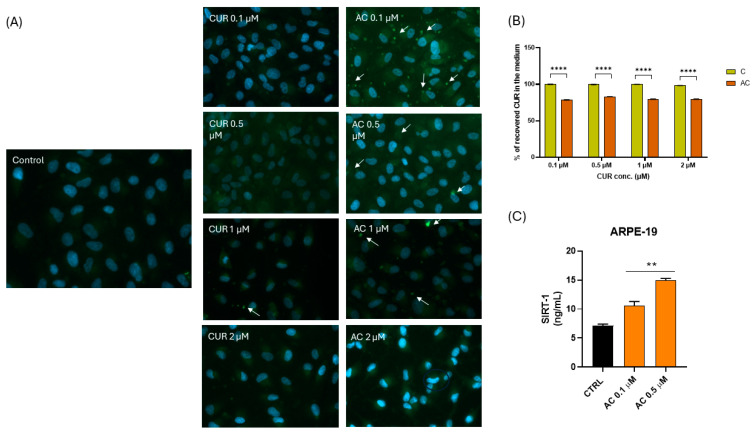
The (**A**) internalization and uptake of CUR (central column): 0.1 µM; 0.5 µM; 1 µM; 2 µM and CUR-loaded SNEDDS (right column): AC 0.1 µM; AC 0.5 µM; AC 1 µM; AC 2 µM into ARPE-19. White arrows point out CUR nanocarriers poutside cells. (**B**) Quantitative evaluation of recovered CUR in medium and not internalized after uptake test (**** *p* < 0.0001 vs. C). (**C**) Assessment of SIRT1 protein expression levels following AC treatment for 24 h at selected concentrations of 0.1 and 0.5 µM (** *p* < 0.005 vs. CTRL).

**Figure 8:**
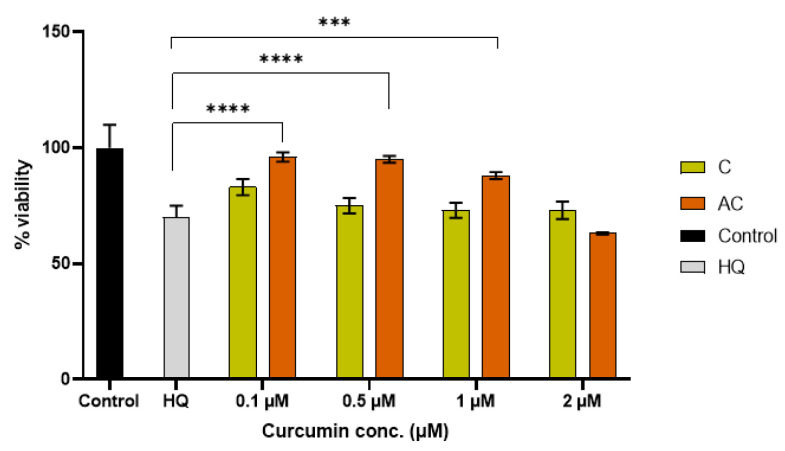
Evaluation of HQ (600 μM) effect on ARPE-19 cell viability and recovery with co-treatment of HQ and SNEDDS loaded with different concentrations of CUR (0.1–2 μM) (**** *p* < 0.0001 vs. HQ; *** *p* < 0.0005 vs. HQ).

Internalization of CUR is a key parameter in this study, as SIRT1 is an intracellular protein, and free CUR without a carrier is unlikely to cross the cell membrane. ARPE-19 cells were exposed to different concentrations of free CUR (0.1–5 μM) and CUR encapsulated in CUR-SNEDDS (0.1–5 μM). A microscopic analysis revealed that cells treated with CUR-SNEDDS exhibited significantly higher fluorescence intensity compared to those treated with free CUR, indicating that the nanosystems were internalized more effectively by the cells than native CUR (Figure 7 and Figure 8). The nuclei are depicted in blue, while prominent yellow spots likely represent oil globules containing CUR. At a concentration of 0.1 μM, free CUR was not detected inside the cells, whereas CUR encapsulated in SNEDDS exhibited pronounced fluorescence, particularly in the nuclei, indicating an intracellular uptake of CUR. This result is particularly noteworthy due to the nuclear localization of SIRT1. A similar trend was observed at concentrations of 0.5 μM and 1 μM, where the fluorescence intensity for CUR-SNEDDS was significantly higher than for free CUR, confirming enhanced uptake. At 5 μM, noticeable fluorescence was observed even with free CUR, likely due to a concentration gradient between the extracellular CUR and the intracellular CUR-poor environment. However, at the same concentration, CUR-SNEDDS-treated cells exhibited decreased cell numbers and altered morphology, indicating a reduced cell viability that is consistent with the results shown in Figure 6B. The encapsulation of CUR in SNEDDS increased its permeability by entrapping it within fine oil droplets dispersed in an aqueous solution. Due to the lipid droplets’ small size, this o/w nanoemulsion is able to enhance permeability and diffusion across the membrane, suggesting that the tiny oil droplets may traverse the cell membrane via the transcellular pathway [29]. Figure 7B shows a quantitative analysis of CUR recovered in the medium after the uptake test, and thus not internalized, comparing the free CUR and the CUR-SNEDDS at equivalent concentrations. Corroborating the figure’s data, around 20% of the CUR was internalized inside the cells at a lower concentration (0.1 μM). At higher concentrations, more CUR was internalized but maintained a similar percentage with respect to its control. Free CUR was recovered at 10% in the medium, revealing no internalization in the cell and only 1% at higher concentrations such as 1 μM and 2 μM, probably for gradient concentration. These results corroborate the enhanced permeability and uptake of CUR-SNEDDS compared to free CUR. To further evaluate CUR-SNEDDS’s ability to induce CUR protein target SIRT1 expression, an ELISA quantitative analysis was performed. Based on the uptake results, the 0.1 and 0.5 μM AC concentrations were selected. After the treatments, the cells were collected and the protein lysates were analyzed for SIRT1 expression. As reported in Figure 7C, the formulation was able to significantly increase SIRT1 protein levels compared to the control, supporting the improvement in CUR bioavailability.

### 2.8. Antioxidant Activity

The effect of free CUR (C) and CUR within SNEDDS (AC) at different concentrations (0.1–5 μM) was tested on ARPE-19 cells previously exposed to 600 μM HQ to investigate a potential protective effect. A significant difference is observed with CUR within SNEDDS at low concentrations. With HQ, there is a decrease in cell viability to 70%, which was reversed up to 96%, 95%, and 88% following AC treatment at concentrations ranging from 0.1 μM to 1 μM. This effect is not evident with free CUR alone, as there is no significant difference compared to the cells exposed to the insult. CUR enters due to the concentration gradient between the outside and the CUR-poor inside of the cell. At high concentrations of CUR that start at 2 μM, CUR within SNEDDS enters massively, likely leading to a cytotoxic effect, which, instead of increasing the cell viability, tends to further decrease it, leading to cellular toxicity. It should be taken into account at this stage that CUR also plays a role as an anticancer agent and cell proliferation blocker at concentrations of 5–10 μM, considering that its IC50 value is approximately 10 μM [30]. Thus, our result corroborates the literature scenario.

## 3. Discussion

The SIRT1 pathway plays a critical role in mitigating inflammation and oxidative stress cascades that are commonly associated with degenerative ocular diseases, including DR. However, SIRT1 expression is downregulated in degenerative and age-related conditions, such as retinopathies and diabetes-related disorders like DR. The development of therapies targeting these pathologies is particularly challenging due to the limitations of conventional treatments and the poor bioavailability of SIRT1 agonists when administered topically. Topical administration, while potentially safer than intravitreal injections, suffers from low drug delivery efficiency [31,32]. Additionally, the intracellular localization of SIRT1 complicates the internalization of therapeutic molecules. CUR, a natural compound with potent anti-inflammatory and antioxidant properties, exerts its effects partially through SIRT1 activation. Despite its therapeutic potential, CUR’s efficacy is hindered by its lipophilic nature, low bioavailability, poor intracellular uptake, susceptibility to photodegradation, and instability in neutral/alkaline aqueous environments. SNEDDS offers a promising strategy to enhance the pharmacokinetic and pharmacodynamic profiles of poorly water-soluble drugs like CUR. These systems form a nanoemulsion upon exposure to the aqueous tear environment after the installation of the anhydrous mixture, making them a potential alternative to traditional ocular suspensions for lipophilic compounds. In our previous work, the SNEDDS formulation was optimized using DoE software to construct ternary phase diagrams and identify the final formulations suitable for topical ocular administration. This approach ensured the development of stable and efficient delivery systems tailored for the enhanced therapeutic outcomes of very lipophilic molecules [22]. To select the most suitable vehicle for CUR delivery, a solubility test was conducted to identify the optimal components for CUR solubilization. It is essential to ensure complete solubilization of the drug in the anhydrous SNEDDS mixture, which comprises oil, surfactant, and co-surfactant and serves as the drug carrier. An extensive preformulation study conducted in our previous work [22] enabled the selection of the optimal parameters, materials, and component ratios that are critical for nanoemulsion formation under specific conditions, including minimal volume for resuspension, a temperature of 37 °C, and physiological pH, which closely mimic the ocular surface environment. Building on this prior research and the solubility studies of CUR, the following material was selected: Capryol^®^ PGMC (hydrophyl–lipophyl balance, HLB 5), chosen for its superior CUR solubilization capacity, high solubility in both the aqueous and lipid phases (outperforming Capryol^®^ 90), low viscosity for ease of handling, and recognized biocompatibility by regulatory authorities. Additionally, it exhibits emulsification properties, facilitates absorption enhancement across biological membranes, and demonstrates robust chemical stability [33]. Tween^®^ 80 (HLB 15.00) was selected for its ability to reduce particle size, attributed to its molecular weight, which is more favorable compared to other polymeric surfactants. The literature indicates that a higher HLB value in the surfactant reduces interfacial energy, promoting the formation of a stable emulsion. Transcutol^®^ P (HLB 4.2) was finally selected as the co-surfactant due to its superior stability, excellent emulsification efficiency (particularly in combination with Tween^®^ 80), and low volatility [34]. The synergistic effects of these components, as indicated by the HLB values and ternary phase diagrams, result in reduced interfacial tension and the formation of a stable nanoemulsion. Based on these findings, a formulation composed of the previously mentioned materials was selected from prior work and already designated as SNEDDS A. The latter was loaded with CUR. A physicochemical characterization of CUR-loaded SNEDDS demonstrated optimal suitability for ocular instillation. Upon reconstitution in a small volume of STF, mimicking the post-instillation environment, no signs of CUR precipitation or destabilization were observed, indicating the effective encapsulation of CUR within the formed globules. Achieving an optimal ratio between surfactant and co-surfactant was identified as a key parameter for ensuring physical stability [35]. Emulsification occurred in under one minute, a critical factor for the rapid formation of a stable nanoemulsion, preventing the drainage of the formulation before effective action [36]. Upon reconstitution, the mixture formed very small, spherical micelles capable of entrapping at least 99% of CUR. Droplet size was a crucial parameter for stability and bioavailability; droplets smaller than 50 nm provided a larger interfacial surface area, facilitating faster absorption and improved bioavailability. According to Puglia et al., nanoparticles of approximately 50 nm can penetrate the posterior segment of the eye via the transscleral pathway following topical instillation [36]. To enhance the electrostatic interactions with the ocular mucus, the neutral charge of effective nanoparticles was reversed by incorporating DDAB, improving the mucoadhesive properties [37]. Mucoadhesive interactions initiate immediately upon contact between positively charged SNEDDS AC+ and mucin dispersion, increasing residence time, potentially reducing administration frequency, and improving patient compliance. Mucoadhesive formulations also enhanced drug penetration into ocular tissues, enabling targeted treatment while minimizing systemic absorption and reducing the risk of systemic side effects. Prolonged contact time and prevention of formulation drainage before emulsion formation were identified as crucial features. As noted by Phan et al., ocular lubricants should exhibit viscosities of at least 10 to 15 mPa·s to enhance retention time on the ocular surface; the viscosity of SNEDDS AC aligns with this recommendation [38]. One major challenge in ocular product formulation is instability and biodegradation at physiological pH. However, our study demonstrated that SNEDDS formulations effectively stabilized CUR, preventing its degradation for up to one week under various conditions by protecting the encapsulated CUR from hydrolysis and biotransformation [39].

Studying the release rate from the delivery vehicle is essential for quality control, predicting in vivo behavior, and elucidating the system’s structure and release mechanism. CUR exhibits a burst release within the first 6 h, a characteristic feature of SNEDDS, which maintains the drug in a dissolved state and shortens the release time [40]. Following this initial phase, the release becomes sustained, a behavior typical of highly lipophilic compounds. This sustained release is likely attributable to interactions between CUR and lipid micelles, as well as saturation of the external medium. When SNEDDSs come into contact with tear fluid, they transform into nanoemulsion droplets that adhere to the lipophilic corneal epithelium. This adherence minimizes formulation drainage and facilitates sustained drug release via the carrier.

The compatibility of the formulation was assessed using two cell lines, namely ARPE-19 and HCE, to evaluate the cytocompatibility in the two primary targets of the prepared formulation: the ocular surface, where SNEDDS reconstitution and nanoemulsion formation occur, and the retina, which is the target tissue of the addressed pathology. AC demonstrated cytocompatibility up to a concentration of 5 μM of CUR. AC demonstrated cytocompatibility up to a concentration of 5 μM of CUR. CUR exhibits a cytotoxic role as an antiproliferative and anticancer agent starting at 5 μM. SNEDDS increases the internalization of CUR from 0.1 μM, which is important for serving as an agonist of intracellular SIRT1 [41]. The internalization of CUR-SNEDDS was more efficient at lower concentrations, as higher CUR concentrations led to cytotoxic effects. Quantitative analysis of the CUR recovered from the medium after uptake, corresponding to non-internalized CUR, showed significant internalization (*** *p* < 0.0001 AC vs. C). Approximately 20% of the CUR was internalized following exposure to CUR-SNEDDS, compared to 0% internalization and 100% of CUR in the medium after exposition of free CUR. The aim of this work has been achieved since the SNEDDS are more effective in terms of drug internalization. The reduced particle size of SNEDDS increases the surface area available for interaction with cellular membranes, enhancing the probability of absorption and facilitating drug entry into cells [42]. Furthermore, Tween^®^ 80 contributes to this process by interacting with lipids in cellular membranes, increasing their permeability and facilitating drug absorption. Similarly, Transcutol^®^ P acts as a penetration enhancer, aiding the passage of molecules across cellular membranes. Together, these absorption enhancers improve CUR uptake into cells. In contrast, free CUR showed slight internalization only at higher concentrations, likely due to a concentration gradient driving transport into cells or interactions between CUR and phospholipid membranes. However, to avoid cytotoxic effects, SNEDDS enable CUR internalization at lower concentrations, sufficient to activate SIRT1, thereby eliminating the need for high doses. Indeed, the protective role of native CUR and CUR-SNEDDS against oxidative stress in ARPE-19 cells was evaluated, establishing an effective dose range for AC between 0.1 and 1 μM [43]. This result further corroborates the uptake results and confirms the SIRT1 induction ability of AC, which is in line with the scenario of SIRT1 agonist activity. At low concentrations, the SIRT1 pathway was most likely activated, protecting retinal cells from oxidative stress.

## 4. Materials and Methods

### 4.1. Materials

CUR (purity 95.0% by HPLC) was produced by Giellepi SpA (Seregno, Italy), kindly gifted by Labomar SpA (Istrana, Italy), and it was used as the active ingredient. Several lipids are used for solubility screening: Mygliol^®^ 812 from IOI Oleo GmbH (Witten, Germany), isopropylmyristate (IPM) from A.C.E.F (Fiorenzuola d’Arda, Italy), Tegin^®^ O, Capryol^®^ PGMC and Capryol^®^ 90 donated by Gattefossé SAS (Saint-Priest, France), and Castor oil^®^ from Sigma (Schnelldorf, Germany). Tween^®^ 80 (Polysorbate 80), Tween^®^ 20, Cremophor EL^®^, and Solutol ^®^HS15 purchased from Merck (Darmstadt, Germany) were used as surfactants. Lastly, Transcutol^®^ P was offered by Gattefossé SAS (Saint-Priest, France) and was used as a co-surfactant. PBS in tablets 7.2–7.6 (1 tablet/200 mL) was purchased from Merck (Darmstadt, Germany). STF was freshly prepared using protocols widely found in the literature.

### 4.2. Excipients Screening

The oil, surfactants, and co-surfactants were selected based on the solubility of CUR and the stability of the formed SNEDDSs. Different types of oils were selected based on biocompatibility and biodegradability: Mygliol^®^ 812, Capryol^®^ PGMC and Capryol^®^ 90, Tegin^®^ O, Isopropylmyristate (IPM), and Castor oil. Five surfactants, including Tween^®^ 20, Tween^®^ 80, Cremophor EL^®^, Solutol ^®^HS15, and Transcutol^®^ P, were selected for the experiment in order to investigate the best materials for CUR-SNEDDS preparation. To 1 mL of each vehicle, an excess of CUR was added separately in glass vials. The mixtures were stirred (350 rpm) using a magnetic stirrer for 24 h. The samples were covered in order to prevent photo-degradation. After that, each dispersion was centrifuged for 30 min (10,000 rpm) at 25.0 ± 1.0 °C to separate the undissolved CUR from the solubilized one. The supernatant was collected in fresh vials; suitable dilutions were made using methanol, followed by the determination of a dissolved CUR concentration using UV–visible spectrophotometer UH5300, Hitachi, Chiyoda, Japan. Quantification of CUR in the samples was obtained by comparing the absorbances found in the supernatant with the calibration line in methanol, which was linear in the concentration range of 1.415–11.32 μg/mL (R^2^ = 0.9983). The absorbance reading occurred at 422 nm.

### 4.3. Preparation of CUR-SNEDDS

CUR-SNEDDS (AC) were prepared using the low-energy method of a simple mixture of the materials and the drug. This approach appears to be simple, scalable, and sustainable due to the absence of organic solvents used in the process. Capryol^®^ PGMC, Tween^®^ 80, and Transcutol^®^ P were used as the oil, surfactant, and co-surfactant, respectively, following the ratio obtained in previous work for optimized formulation [22] and reported in Table 2. Each component was weighed and mixed under agitation at r.t, typically using a magnetic stirrer operating at approximately 100–150 rpm until the blend was fully homogenized. Once a visually uniform mixture was achieved, CUR was added in precisely measured quantities. The formulation was then subjected to further agitation to ensure a complete solubilization of the active ingredient. Unloaded SNEDDS (A) were prepared following the same procedure, omitting the addition of CUR.

### 4.4. Physicochemical Characterization and Morphology

Before characterization, the formulation was reconstituted at 1 to 10 (*v*/*v*) with STF or PBS. Agitation at 35 rpm was employed to simulate the spontaneous emulsification process at the administration site (ocular surface) and to assess the time required for complete homogeneous emulsification.

#### 4.4.1. Globule Size, Polydispersity Index (PDI), and Zeta Potential (ZP)

Particle size characterization is a crucial assessment in the development of SNEDDS, as particle size can influence both in vitro properties (dissolution and stability) and in vivo performance (drug absorption). Globule size, PDI, and ZP were derived by PCS (photon correlation spectroscopy) using the Zetasizer Nano ZS90 (Malvern Panalytical, Malvern, UK) at a 90° angle of detection and 25 °C with a 4 mW He-Ne laser operating at 633 nm. All measurements were performed in triplicate, and the results were expressed as the mean ± standard deviation (SD).

#### 4.4.2. Morphology

The Particle Matrix ZetaView^®^ x30 NTA system (Particle Matrix GmbH, Ammersee, Germany) was used to analyze the morphology of particles. Prior to analysis, the system underwent calibration, auto-alignment, and focusing procedures using a 100 nm polystyrene (PS) standard diluted 250.000 times. Subsequently, the sample containing SNEDDS was prepared and loaded into the chamber using a 1mL syringe. A laser beam was then directed through the sample, causing the nanoparticles to scatter light. The scattered light was captured by a high-resolution camera.

#### 4.4.3. Clarity, pH, Viscosity, and Emulsification Time

The clarity of the CUR-SNEDDS was investigated by measuring the transmittance % at 650 nm using a UV–visible spectrophotometer (UH5300, Hitachi, Chiyoda, Japan) and two cuvettes filled with water (transmittance of 100%) as references. Rheological properties of CUR-SNEDDS were investigated using a rotational rheometer (HAAKE™ MARS™, Thermo Fisher Scientific, Waltham, MA, USA) with a parallel plate geometry (P35/Ti, Thermo Fisher Scientific, USA) of 35 mm diameter. The shear rate was tested at 25 °C in a range between 0.01 and 700 (s-1). The emulsification time is the time (in seconds) required to achieve a clear emulsion without visible particles in the suspension, recorded immediately after the reconstitution and agitation at 35 rpm. All analyses were performed in triplicate and expressed as the mean ± SD. The pH of CUR-SNEDDS formulations was measured in triplicate at 25 °C by a pH meter (Mettler Toledo, Columbus, OH, USA) after a reconstitution (1:10 *v*/*v*) with STF. The instrument was calibrated using standard Mettler Toledo buffer solutions (pH 4.01 ± 0.02; 7.00 ± 0.02, and 10.00 ± 0.02; slope 99.8%).

### 4.5. Entrapment Efficiency (EE) % and In Vitro Release

The EE% was measured by the UV method following the protocol described by Zingale et al. [22] for sample preparation. Accordingly, the CUR-SNEDDS was centrifuged for 30 min at r.t and at 10,000 rpm. The supernatant was suitably diluted with methanol and analyzed by UV spectrophotometry. The amount of CUR in the sample was determined from the peak area correlated with the standard curve obtained from high-performance liquid chromatography (HPLC) analysis described in the following Section 4.6. The supernatants were analyzed in triplicate, and the CUR encapsulation efficiency was calculated by knowing the total amount of drug used to prepare the systems, according to the following equation:

$$EE\%=\frac{\mu g\;of\;tCUR-\mu g\;of\;sCUR}{\mu g\;of\;tCUR}\times 100$$

where tCUR corresponds to the total CUR added in the initial preparation and sCUR is the amount of CUR found in the supernatant.

The in vitro release test of CUR was conducted to simulate physiological conditions. The sample, 0.1 mL of AC (reconstituted 1:10 in freshly prepared PBS), was placed inside a Slide-A-Lyzer™ Dialysis cassette (Thermo Fisher Scientific Inc., Waltham, MA, USA) with a 3.5K MWCO (molecular weight cut-off). These dialysis cassettes were then positioned within individual Eppendorf^®^ tubes containing 1.6 mL of medium (60% PBS (1M, pH 7.45), 30% MeOH, and 10% Tween 80). The experiment was conducted using a shaking platform (Titramax 1000, Heidolph Scientific Products GmbH, Schwabach, Germany) at 100 rpm at a temperature of 37.0 °C ± 1.0 °C. Following pre-established time intervals (30 min, 1 h, 2 h, 4 h, 6 h, 24 h, and 48 h), aliquots were withdrawn from the released medium and replaced with PBS, which was also kept within the shaking machine at the same temperature. The samples were analyzed in triplicate using an HPLC method described in the section below.

### 4.6. HPLC Method

The amount of CUR in both the EE% and the in vitro release experiments was determined by high-performance liquid chromatography (HPLC, Agilent 1200 series, Santa Clara, CA, USA) with a standard curve (y = 58.447x + 5.3164; R^2^ = 0.9998). HPLC analysis was performed on a reversed-phase C18 column (4.6 mm × 150 mm, 5 μm, ZORBAX Eclipse SB-C18) at 25 °C. The mobile phase was composed of water with 4% acetic acid and acetonitrile (44:56, *v*/*v*), filtered through a 0.22 μm Milli-pore filter and degassed prior to use. The flow rate was 0.850 mL/min, and the eluent was detected by a DAD detector at 430 nm.

### 4.7. Stability Studies

The stability of CUR unencapsulated (C) and inside SNEDDS (AC) was assessed under various storage conditions over different intervals of time. Two samples were subjected to stability studies, with AC and C consisting of raw CUR in a PBS solution with 10% MeOH to enhance the CUR dissolution. The preparation of the sample AC followed the procedure described in Section 4.3. The sample was divided into four glass vials, with each set of vials being stored under different conditions, namely at 4 °C dark, 25 °C dark, 25 °C light, and 40 °C dark, to assess the typical storage conditions and investigate possible protection from degradation phenomena. Sample C was prepared differently. A CUR solution was prepared in a plastic tube by adding 10 mL of a stock solution (1:10 ratio) of methanol and PBS (1 M, pH 7.4) to CUR. The mixture was then subjected to magnetic stirring at 350 rpm for 20 min. After stirring, the tube was centrifuged at 13,000 rpm for 20 min at 25 °C. The supernatant was collected and divided into four glass vials, each representing a different storage condition compared to the AC sample. The stability of the samples was evaluated at 6 h, 24 h, 48 h, and 7 days to monitor potential changes in the CUR concentration under different storage conditions. HPLC analysis was employed to detect any variations in absorbance.

### 4.8. Cationic CUR-SNEDDS

Cationic CUR-SNEDDS (AC+) was prepared as reported in Section 4.3, adding a small amount of DDAB (0.25%*w*/*v* in the 1:10 reconstitution). AC+ were subjected to physicochemical characterization following Section 4.4. The primary objective is to enhance the retention time on the ocular surface following instillation and before the formulation is eliminated through tear drainage. With this aim, a mucoadhesion investigation was performed, comparing the interaction with mucin of AC and AC+. A mucin suspension (Muc) was prepared at the concentration of 0.1% *w*/*v* in freshly prepared TRIS buffer, which was stirred for 24 h, referred to as Muc + TRIS henceforth. Subsequently, the suspension Muc + Tris was added in a ratio of 1:1 to AC and AC+, forming two other samples, namely AC + Muc and AC (+) + Muc, respectively. The samples were then incubated in a thermostatic oven at 37 °C and analyzed at various time intervals (0, 30, 60, and 180 min) using UV spectrophotometry, and the absorbance at 650 nm and the ZP values were measured. A cuvette with TRIS buffer was used as a reference for UV analysis. Each analysis was performed in triplicate.

### 4.9. Cytocompatibility Study of CUR and CUR-SNEDDS

#### 4.9.1. Human Corneal Epithelium (HCE) Cells

HCE cells were cultured at 37 °C and 5% CO_2_ using a growth medium consisting of 1:1 DMEM/F12 (Gibco by Life Technologies Limited, Paisley, UK), 15% FBS (fetal bovine serum; Gibco by Life Technologies Limited, Paisley, UK), 100 U/mL penicillin, 0.1 mg/mL streptomycin (penicillin/streptomycin solution from EuroClone spa, Pero, Italy), 0.3 mg/mL L-glutamine (EuroClone spa, Pero, Italy), 10 ng/mL EGF (Recombinant Human Epidermal Growth Factor; Gibco by Life Technologies Corporation, Carlsbad, CA, USA), 5 μg/mL human recombinant insulin (Gibco by Life Technologies Corporation, Grand Island, NY, USA), and 0.5% sterile filtrated DMSO (Sigma-Aldrich, St. Louis, MO, USA), 0.1 μg/mL cholera toxin (Sigma-Aldrich, St. Louis, MO, USA). The fresh growth medium was changed for the cells every 2–3 days. When reaching a 75–80% confluence, the cells were either subcultured 1:5–1:30 or seeded for the experiment. Upon confluence, the cells were washed with 1xDPBS (Gibco by Life Technologies Limited, Paisley, UK) and detached with 0.05% trypsin–EDTA (Gibco by Life Technologies Limited, Paisley, UK). The cytocompatibility test was conducted on 96-well plates with approximately 2 × 10^4^ cells/well. The experiments were performed 24 h after seeding using an FBS-free medium. To determine the toxicity of CUR (Sigma Chemical, St. Louis, MO, USA, dissolved in DMF 0.1%), the cells were rinsed once with serum-free medium to remove FBS residues and exposed to 0.1–5 μM concentrations of SNEDD-CUR and CUR native diluted in a medium. The cells were exposed for 3 h before the cell viability tests. Cell viability was assessed using the 3-(4,5-dimethyl-thiazol-2-yl)-2,5-diphenyltetrazolium bromide (MTT) assay. Cells were assessed with microscopy before the viability test to confirm the correlation of the absorbance readings from the MTT assay.

#### 4.9.2. Human Retinal Pigment Epithelia (ARPE-19) Cells

A similar protocol to the one described above was used to test the cytotoxicity of native CUR and CUR-SNEDD formulation on ARPE-19 cells. The ARPE-19 cell line obtained from the American Type Culture Collection (ATCC, Manassas, VA, USA) was used in the study. Cells were cultured in Dulbecco’s modified eagle medium/nutrition mix F-12 (1:1; Gibco, Paisley, UK) supplemented with 10% fetal bovine serum (FBS; Hyclone, Logan, UT, USA), 100 U/mL penicillin (Lonza, Walkersville, MD), and 2 mM L-glutamine (Lonza, Walkersville, MD, USA). The cells were grown at 37 °C in a humidified 5% CO_2_ atmosphere. The experiments were performed 24 h after seeding using an FBS-free medium. To determine the toxicity of CUR (Sigma Chemical, St. Louis, MO, USA, dissolved in DMF 0.1%), the cells were rinsed once with serum-free medium to remove FBS residues and exposed to 0.1–5 μM concentrations of SNEDD-CUR and CUR native diluted in a medium. The cells were exposed for 3 h before the cell viability tests. Cell viability was assessed using the MTT assay. Cells were assessed with microscopy before the viability test to confirm the correlation of the absorbance readings from the MTT assay.

### 4.10. SIRT1 Expression Levels in ARPE-19 Cells

SIRT1 levels were determined using a Simple Step ELISA (ab171573, Abcam Ltd., Cambrodge, UK). Cells were treated with selected concentrations of SNEDD-CUR (0.1–0.5 μM) for 24 h, and the samples were processed following the manufacturer’s instructions. A microplate reader was used to measure absorbance (OD) at λ = 450 nm. All samples were measured in triplicate, and the results are expressed as ng/mL.

### 4.11. Cell Viability in Simulated Oxidative Stress Conditions

To assess the effective and protective dose of CUR-SNEDDS against oxidative stress, the ARPE-19 cells were rinsed once with serum-free medium and incubated in CUR-SNEDDS (0.1–2 μM) for 3 h. Cells were rinsed once using an FBS-free medium to remove any residual CUR-SNEDDS. Afterward, the oxidative stress-provoking agent, HQ (Sigma-Aldrich), was added. The cells were exposed to 600 μM HQ for 2 h to achieve 60–70% viability, as determined in the control samples [44]. Cell viability was assessed using the MTT assay. Cells were assessed with microscopy before the viability test to confirm the correlation of the absorbance readings from the MTT assay.

### 4.12. Cell-Uptake Investigation in Retinal Cell

ARPE-19 cells were seeded on acid-etched coverslips placed into a 6-well plate with a seeding density of 850,000 cells/well and incubated at 37 °C for 24 h. The cells were then incubated with media containing AC at the same concentration that was used for the cytotoxicity experiment. After 3 h of exposure to the AC and C formulations, the coverslips were removed, washed twice with PBS, and fixed with 4% paraformaldehyde (PFA) for 15 min. Subsequently, the PFA was removed, followed by two washes with PBS. The dried coverslips were then mounted on a glass slide, placed upside down onto a droplet of NucBlue™ Live ReadyProbes™ Reagent (Hoechst 33342) (Thermo Fisher Scientific Inc., Waltham, MA, USA), a blue fluorescent dye specifically staining cell nuclei, and left to dry overnight in the absence of light to prevent CUR photobleaching. Cell imaging was conducted using a ZEISS Axio Imager M2 fluorescent microscope (Carl Zeiss Microscopy Deutschland GmbH, Oberkochen, Germany). The in-direct quantification of CUR uptake was assessed using the same cell line, seeded into 96-well plates at approximately 3 × 10⁴ cells/well. The experiments were conducted 24 h after seeding, using an FBS-free medium. The cells were rinsed once with a serum-free medium to remove the FBS residues and then exposed to 0.1–2 μM concentrations of SNEDD-CUR diluted in a medium. After a 3 h exposure period, the medium was removed and collected into another 96-well plate. The concentration of CUR remaining in the medium was quantified using spectrophotometry by measuring the absorbance at 450 nm. Quantification was expressed in % of CUR recovered in the medium with respect to the initial concentration used for the test.

### 4.13. Statistical Analysis

The mucoadhesion data and in vitro cellular studies are representative of three independent experiments, with the statistical analysis conducted using two-way ANOVA. The statistical analysis was performed using GraphPad Prism 9.5.0 (GraphPad Software, Inc., San Diego, CA, USA). Šídák’s multiple comparisons test was employed for the mucoadhesion studies, antioxidant activity in ARPE-19 cells, and cytocompatibility assessed by the MTT assay in ARPE-19 cells. For the MTT assay in HCE cells, Dunnett’s multiple comparisons test was used. Statistical analysis for stability studies was performed using Tukey’s multiple comparisons test. The results are representative of at least three independent experiments, with values expressed as a percentage of the control. Statistical significance is indicated as * *p* < 0.05, ** *p* < 0.005, *** *p* < 0.0005, and **** *p* < 0.0001.

## 5. Conclusions and Future Directions

As a potential delivery system for enhancing CUR ocular absorption, bioavailability, and retinal protection, SNEDDS loaded with CUR can be synthesized using a simple, low-energy, and solvent-free method. The resulting formulation demonstrated reduced particle size (<50 nm), clarity (~100% of T%), high CUR solubilizing capability and stability at physiological pH, and improved cellular permeation capacity. However, normal and accelerated stability studies will be conducted in the future to evaluate CUR stability within the anhydrous SNEDDS under different storage conditions of temperature and humidity over an extended period. A slow release was reported for 48 h, but in the first hours of the release test, the SNEDDSs showed an increase in dissolution, permeability, and release compared to the free CUR. CUR-SNEDDS allowed for an increase in the internalization in retinal cells and promoted SIRT1 expression at a lower concentration. Additionally, CUR-SNEDDSs exhibited cytoprotective effects in an in vitro HQ-induced oxidative stress model on ARPE-19 cells. Given that the formulation demonstrated optimal characteristics for topical ocular administration, including favorable physicochemical parameters, appropriate pH, and corneal cytocompatibility, SNEDDS represents a promising strategy to improve the solubility, bioavailability, and stability of molecules with poor solubility, limited bioavailability, and instability in physiological environments. However, a significant challenge is evaluating the effective delivery of CUR to deeper tissues, as it must overcome several barriers within the eye. Topical administration is considered the safest route but also the most challenging for degenerative eye diseases such as retinopathies. Therefore, future studies will focus on in vivo investigations to assess retina achievement following CUR-SNEDDS instillation. Building on evidence that low concentrations of CUR-SNEDDS enhance SIRT1 expression and protect retinal cells from oxidative stress, future studies will focus on translating these findings to in vivo models. Specifically, CUR-SNEDDS could be evaluated in an in vitro oxidative stress model induced by high glucose levels. This approach will enable a thorough assessment of its therapeutic efficacy in an in vivo model of DR, following a comprehensive investigation of its delivery to the retina. SNEDDS represents a novel approach that warrants more attention in the ocular field as an advanced in situ generator of a nanoemulsion.

## Figures and Tables

**Table 1:** Physicochemical characterization of A, AC, and AC+ in terms of globule size, PDI, ZP, Transmittance, and time of emulsification.

Sample	Size (nm) ± SD	PDI ± SD	ZP ± SD	Transmittance (%)	Time of Emulsification (s)
A	13.26 ± 0.07	0.105 ± 0.017	−4.02 ± 1.02	100	~12.04
AC	13.44 ± 0.19	0.095 ± 0.021	−4.12 ± 0.88	99	~31.22
AC+	14.02 ± 0.22	0.112 ± 0.032	+2.92 ± 0.09	99	~32.44

**Table 2:** Composition in terms of oil, surfactant, and cosurfactant of SNEDDS (A) and CUR-SNEDDS (AC).

Sample	Capryol^®^ PGMC (%*w*/*w*)	Tween ^®^ 80 (%*w*/*w*)	Transcutol ^®^ P (%*w*/*w*)	CUR (mg/mL)
A	15.04	55.18	28.21	
AC	15.04	55.18	28.21	1

## Data Availability

The original contributions presented in this study are included in the article/Supplementary Materials. Further inquiries can be directed to the corresponding authors.

## Supplementary Materials

The following supporting information can be downloaded at: https://www.mdpi.com/article/10.3390/ph18020265/s1, Table S1. CUR (%) recovered after exposition of free CUR in PBS and CUR-SNEDDS reconstituted in PBS at different storage conditions of temperature and light/dark for up to 168 h.

## Abbreviations

AMD	Age-related macular degeneration
AMPK	AMP-activated protein kinase
ARPE-19	Retinal pigment epithelial cells
CUR	Curcumin
DDAB	Didodecyldimethylammonium bromide
DoE	Design of experiments
DR	Diabetic retinopathy
EE	Entrapment efficiency
GLP-1	Glucagon-like peptide 1
HCE	Human corneal epithelial cells
HLB	Hydrophyl-lipophyl balance
HO-1	Heme oxygenase-1
HPLC	High-performance liquid chromatography
HQ	Hydroquinone
NF-κB	Nuclear factor kappa–light-chain enhancer of activated B cells
PBS	Phosphate buffer saline
PDI	Polydispersity index
PPAR-γ	Proliferator-activated receptor-γ
ROS	Reactive oxygen species
R.T	Room temperature
SIRT1	Sirtuin-1
SOD	Superoxide dismutase
SNEDDS	Self-nanoemulsifying drug delivery systems
STF	Simulated tear fluid
TNFα	Tumor necrosis factor
VEGF	Vascular endothelial growth factor
ZP	Zeta potential
