# Supplementary material for: Protective Role and Enhanced Intracellular Uptake of Curcumin in Retinal Cells Using Self-Emulsifying Drug Delivery Systems (SNEDDS)
Source: Pharmaceuticals (Basel). 2025 Feb 17;18(2):265. doi: 10.3390/ph18020265 (PMC11859040; doi:10.3390/ph18020265)
Supplement: Supplementary file 1 [file pharmaceuticals-18-00265-s001.zip › pharmaceuticals-3447730-supplementary.pdf]

Table S1. CUR (%) recovered after exposition of free CUR in PBS and CUR-SNEDDS reconstituted in PBS at different storage conditions of temperature and light/dark for up to 168 h.

**% of recovered curcumin**					
Sample	Storage condition	Time (h)			
		6	24	48	168
C	4°C	57.2	22.8	0	0
	25°C Dark	9.7	0	0	0
	25°C Light	0	0	0	0
	40°C	0	0	0	0
AC	4°C	83.5	83	79.2	76.6
	25°C Dark	80.2	76.1	72	38
	25°C Light	63.9	9.4	0	0
	40°C	88.2	68.6	37.1	0
